# No association between HPV positive breast cancer and expression of human papilloma viral transcripts

**DOI:** 10.1038/srep18081

**Published:** 2015-12-14

**Authors:** Orla M. Gannon, Annika Antonsson, Michael Milevskiy, Melissa A. Brown, Nicholas A. Saunders, Ian C. Bennett

**Affiliations:** 1 The University of Queensland Diamantina Institute, The University of Queensland, Translational Research Institute, Brisbane, QLD; 2 Department of Population Health, QIMR Berghofer Medical Research Institute, Herston, QLD; 3 School of Chemistry and Molecular Biosciences, The University of Queensland, Brisbane, QLD; 4 Faculty of Medicine and Biomedical Sciences, The University of Queensland, Brisbane, QLD; 5 Princess Alexandra Hospital, University of Queensland Department of Surgery, Brisbane, QLD; 6 Wesley Private Hospital, Auchenflower, Brisbane, QLD

## Abstract

Infectious agents are thought to be responsible for approximately 16% of cancers worldwide, however there are mixed reports in the literature as to the prevalence and potential pathogenicity of viruses in breast cancer. Furthermore, most studies to date have focused primarily on viral DNA rather than the expression of viral transcripts. We screened a large cohort of fresh frozen breast cancer and normal breast tissue specimens collected from patients in Australia for the presence of human papilloma virus (HPV) DNA, with an overall prevalence of HPV of 16% and 10% in malignant and non-malignant tissue respectively. Samples that were positive for HPV DNA by nested PCR were screened by RNA-sequencing for the presence of transcripts of viral origin, using three different bioinformatic pipelines. We did not find any evidence for HPV or other viral transcripts in HPV DNA positive samples. In addition, we also screened publicly available breast RNA-seq data sets for the presence of viral transcripts and did not find any evidence for the expression of viral transcripts (HPV or otherwise) in other data sets. This data suggests that transcription of viral genomes is unlikely to be a significant factor in breast cancer pathogenesis.

Worldwide, breast cancer is the most common cancer affecting women. There were 1.7 million new cases reported in 2012, representing approximately 12% of all new cancer cases and 25% of all cancers in women. Although incidence trends for older women have recently stabilized, younger women are experiencing a rising incidence^1^. Despite the efforts of large scale multinational consortia no clear genomic feature(s) can explain the high incidence of breast cancer. For example, even within familial breast cancer cohorts we can only identify evidence of genetic variants that predispose to breast cancer in 30 percent of cases^2^. Furthermore, there are significant population differences in the incidence of breast cancer that are not explained by ethnicity alone^3^,^4^. This means that the majority of breast cancers occur sporadically, without evidence for a heritable genetic, transcriptomic, or epigenetic cause. These factors, combined with evidence that a high proportion (estimated at 16%) of other cancers are caused by infectious agents^5^, led to the hypothesis that oncogenic viruses may be an etiologic factor in sporadic breast cancer. However, despite many attempts the association of viruses such as mouse mammary tumour virus (MMTV)^6^, herpes viruses^7^, Epstein-Barr virus (EBV)^8^, cytomegalovirus^9^ and human papilloma virus (HPV)^10^ with normal and malignant breast tissue remains in question.

The lack of clarity about a viral association with breast cancer is, in part, attributable to the limits of the technologies used. Using next generation sequencing (NGS) techniques it has become possible to examine deep sequencing data for the presence of pathogenic nucleic acids. An early, successful example of this approach is the identification of a novel polyomavirus, Merkel Cell Polyomavirus^11^ in Merkel Cell carcinoma. Subsequently, similar studies have attempted to identify viruses in deep sequencing datasets in a variety of cancer types using different bioinformatic pipelines^12^,^13^,^14^,^15^. Using this approach it has been possible to identify a novel virus in organ transplant recipients^16^, HPV in squamous cell carcinoma of the head and neck^17^, hepatitis B virus in hepatocellular carcinoma^18^ and Epstein Barr virus in gastric carcinoma^17^. However, to date, NGS approaches have failed to detect the expression of viral RNA (transcripts) in breast adenocarcinoma^17^,^19^,^20^. Moreover, only one study has examined whether a breast cancer sample that is positive for HPV DNA is also positive for HPV transcript^19^. This study failed to find evidence of HPV transcript in the two HPV positive breast cancer samples examined. However, the low HPV DNA prevalence reported by Fimereli *et al.*^19^ differs to the majority of published PCR-based studies in which HPV positivity is greater^10^. This highlights the “playoff” between sensitivity achievable with PCR-based strategies (high sensitivity) and NGS data (lower sensitivity with conventional coverage) against the potential for false positive results (PCR > NGS).

In this study we have interrogated fresh breast cancer tissue using degenerate HPV primer pairs against HPV DNA. By using this approach we have biased our initial screen to detect even very low copy number HPV DNA. Samples which were positive for HPV DNA by nested PCR underwent massively parallel deep sequencing, followed by bioinformatic analysis to determine whether any viral transcripts (HPV or otherwise) were present. Three different bioinformatic pipelines which can identify pathogenic nucleic acids in next generation sequencing data were utilized. Using this highly sensitive approach, we failed to find any evidence for expression of HPV or other viral transcripts in breast cancer samples, even in samples which had detectable HPV DNA.

## Results

### HPV detection by nested PCR

We extracted genomic DNA from 80 breast cancer samples and 10 normal breast tissues and confirmed their integrity by PCR for a 260 bp region on chromosome 1 (S100A8). All samples had readily amplifiable DNA. Samples were then subjected to three repeats of nested PCR analysis using MY09/MY11^21^ and GP5+/6+ primers^22^. Genomic DNA isolated from HeLa cells (infected with HPV18) were used as a positive control, and genomic DNA isolated from HPV negative SCC25 squamous cell carcinoma cell line was a negative control.

HPV positivity was declared when more than one PCR reaction was positive in the second nested PCR. Based on this criteria 1 of the 10 (10%) normal tissue specimens was positive for HPV in the GP5+/6+ PCR and 13 of 80 (16%) breast cancer specimens were positive for HPV. The rates of HPV DNA detected by PCR between benign and malignant tissue specimens was not statistically significant (p = 0.6072, Chi Squared test). There was no statistically significant association between HPV positivity and any clinical or histopathological features (p > 0.05; Table 1). Interestingly, one patient had two separate malignancies over the course of the study (2007 left breast, 2012 right breast), and only the 2007 specimen was positive for HPV DNA.

### HPV RNA detection in HPV DNA positive samples

Total RNA was isolated from five breast cancer samples which were positive for HPV DNA (Table 2, samples in bold), and from HeLa cells. The remaining breast cancer samples did not have RNA of suitable quality for RNA-sequencing due to degradation of RNA from long-term (6–9 years) fresh-frozen tissue storage. Total RNA was depleted of rRNA and tRNA and next generation sequencing libraries were generated and sequenced on the Illumina HiSeq 2500 platform.

The positive control library generated from HeLa cells detected HPV18 viral transcripts, with 34000 sequencing reads attributable to HPV E6 and E7 genes, for a read proportion per million (ppm) of 850, which is comparable to other reports^20^ (Table 2). We next analyzed the five breast cancer RNAseq datasets (Table 2), sequenced to an average of 40 million sequencing reads per sample, using three different bioinformatic pipelines: RINS^13^, READSCAN^15^ and VirusFinder 2.0^14^. In no instance did we find any evidence of HPV, or other viral transcripts, in the samples that were positive for HPV DNA.

### Bioinformatic Analysis for viral RNA in next generation sequencing datasets

It is known that there are geographical variations in breast cancer incidence and that the prevalence of viral transcripts may be low; thus we extended our study to include publicly available breast cancer RNA-seq datasets available from The Cancer Genome Atlas (TCGA) and a cohort of triple negative breast cancer (TNBC)^23^. This also served to validate our findings in an independent data set. We again validated the three different bioinformatic pipelines (RINS, Readscan and VirusFinder 2.0) using publicly available RNA sequencing data for tumour and normal matched tissue from HPV positive oral cavity SCC, EBV infected lymphoma cells, HPV infected HeLa cervical cancer cells and HIV infected T cells (Table 3). As anticipated, viral RNA was detected in these positive control samples (Table 3). We also screened 53 publicly available breast cancer and 10 normal breast tissue RNA seq datasets for the presence of any viral sequences. The cohort of RNA-seq datasets we examined were from triple negative breast cancers (TNBC) and hormone receptor positive breast cancers. In line with other studies^17^,^19^,^20^, and the results presented in this manuscript, no viral transcripts were detected in any of these sequence sets. (Supplementary Table 1).

## Discussion

To our knowledge, this is the first study designed to address the question of whether viral DNA detected in breast tumours are associated with the expression of viral transcripts. To do this we have used a highly sensitive PCR approach to identify HPV DNA positive tissues even at very low copy number. This is used to stratify positive patients for RNA-seq analysis for HPV transcripts. In addition, we used rRNA depleted libraries to allow for the detection of both polyadenylated and non-polyadenylated viral transcripts and expanded our study to interrogate databases for any known human viral transcripts. Using this stringent approach we find no evidence of active viral (HPV and non-HPV) transcription within human breast tumour tissue. Our analysis of RNA sequencing data in HPV-DNA positive breast cancer extends other studies by examining greater numbers of breast cancer samples without prior knowledge of HPV DNA positivity^17^,^19^,^20^ and reaches the same conclusion.

Whilst we find that approximately 16% of breast tumour tissues have HPV DNA, we did not find evidence for viral transcription within those samples which were positive for HPV DNA. The most reasonable conclusion to draw from this is that the viral genomes are not being transcribed and hence are functionally inactive and not able to contribute to oncogenesis. It should be noted that the low copy number for HPV DNA detected could be attributable to an association with nonmalignant cells such as white blood cells since HPV can be detected in peripheral blood mononuclear cells, dendritic cells, B cells and neutrophils^24^, or alternatively the virus may have transited via the ducts and be a bystander but not active in carcinogenesis. Whilst nested PCR may detect viral DNA from a small proportion of cells, the sensitivity of RNAseq analysis is for 10 million sequencing reads, there is a 99.99% probability of detecting at least one viral read if every cell is infected and the viral transcript is present with a frequency of 0.0001% (i.e. 1 transcript per million reads)^17^,^25^. Whilst we cannot deny that higher depth sequencing (i.e. the 100–150 million reads per sample as per TCGA) may yield a higher frequency of HPV genome detection this would also increase the likelihood that positivity was associated with the blood cell elements rather than the malignant tissue compartment.

The literature describes a wide range of oncogenic HPV DNA positivity in breast cancer– from 0%^26^, to 86%^27^ (reviewed in^10^). Several factors may contribute to this variable prevalence, such as differences in sampling populations, different assay sensitivities or potential sample contamination. Interestingly, a recent publication highlighted the prevalence of sample contamination, even in a well run high throughput sequencing facility, with HPV-18 RNA from Hela cells detected in TCGA RNA sequencing data^28^. Indeed, when highly sensitive assays such as nested PCR are utilized, careful controls and experimental procedures must be utilized to ensure that samples do not become contaminated by extraneous HPV DNA.

Whilst it could be argued that we only assayed for HPV transcripts in a small number of HPV DNA positive samples (n = 5) this is sufficient to exclude HPV transcription as a common event in HPV DNA positive specimens. This is also supported by a previous study which also failed to detect oncongenic HPV or other viral RNA in 810 breast cancer cases and 104 normal tissues sequenced at 2–3 fold greater depth than in this study^20^. Even if one assumes that only 10% of the 810 breast cancer samples studied by *Tang et al.* were HPV positive, this still indicates that the vast majority of breast cancer tissue that are positive for HPV DNA fail to make transcripts and thus we conclude that HPV does not play a role as an etiological factor in most breast tumours. Careful analysis of the supplementary data from *Tang et al.* (2014) shows that a very small proportion of the malignant and non-malignant tissue (1.2% and 0.96% respectively) had an extremely low level of HPV-18 (8 reads from 169 million) reads which is well below any reasonable threshold for disease association; furthermore the prevalence is the same in malignant and non-malignant tissues. However, it must also be conceded that no definitive ‘cut off’ for disease causation has been fully accepted for next generation sequencing data to date. In this regard we note that recent reports have shown that the APOCEB3 enzyme, which is highly expressed in breast cancer^29^ and can be regulated the HPV E6 oncogene^30^,^31^, can induce genetic instability and increase breast cancer risk^29^. However, given that we do not find any evidence of HPV oncogene expression in the samples, it would seem unlikely that APOECB3 upregulation is HPV-mediated.

Nonetheless, the effects of the recent introduction of HPV vaccination programmes will provide an interesting epidemiological perspective on the possible aetiology of HPV in breast cancer, although it may be decades until a cause and effect phenomenon can be identified. Similarly, a recent epidemiological study showed that individuals with a compromised immune system have an increased rate of virally-mediated cancers such as Kaposi’s sarcoma and cervical cancer; whereas the incidence of breast cancer is not increased^32^, again supporting our postulate that breast cancer is unlikely to have a viral aetiology.

The only caveats to our conclusion that HPV transcripts do not contribute to breast tumour development are i) that HPV could contribute to rare breast cancer subtypes which were poorly represented in our sample set, ii) that our RNA-seq analysis was unable to detect viral transcripts with a sequence that is greater than 50% divergent from a virus in the reference database (e.g. for RINS^13^), or a virus which is present at very low levels (i.e. less than 0.1–1 copies per cell) or, iii) that the carcinogenic action of a virus acts only at the initiation stage before it is cleared; i.e. the “hit and run” phenomenon seen with bovine papillomavirus (BPV) in oesophageal cancer in cattle^33^. However, notwithstanding these caveats, our work strongly suggests that HPV, or other known viruses, are not expressed in human breast cancer at detectable levels and are unlikely to be a significant aetiological factor in breast carcinogenesis in humans.

## Methods

### Sample Collection

80 breast cancer tissue specimens and 10 non-malignant (from patients with benign breast disease) specimens were aseptically collected by one surgical team. The sample was placed into a sterile tube and transported to a tissue bank, snap frozen and stored at −80 °C. Clinicopathological features, including receptor positivity, were accessed from medical records. This study was approved by the institutional ethics committee and all studies were performed in accordance with the approved protocols. All patients provided written, informed consent for tissue collection for the purposes of research.

### Tissue Culture

HeLa cells were a kind gift from Nigel McMillan (Griffith University, QLD, Australia), were used within 6 months of passaging from receipt from ATCC and were maintained in Dulbecco’s modified Eagle’s medium (Invitrogen, Scoresby, VIC) supplemented with 10% fetal calf serum (GIBCO, Scoresby, VIC), 100 units/mL penicillin G, 100 μg/mL streptomycin sulfate and 0.29 mg/mL L-Glutamine (Invitrogen). SCC25 cells were maintained as per^34^ and were verified by STR genotyping.

### DNA isolation

Using aseptic techniques, a section of frozen breast cancer tissue was isolated using a sterile tissue culture dish (Corning, Murrarie, QLD, Australia) and a sterile scalpel. For cell lines, the cells were released from the tissue culture vessel with trypsin to isolate a cell pellet. DNA was isolated with the Isolate II DNA Isolation Kit (Bioline, Alexandria NSW, Australia) as per the manufacturer’s instructions. DNA concentration was determined using the NanoDrop spectrophotometer (Thermo Scientific, Scoresby, VIC, Australia) and stocks of DNA were made at 10 ng/μL for analysis by PCR.

### RNA isolation

Tissue samples were homogenized using the gentleMACS Octo Dissociator (Miltenyi Biotec, Macquarie Park, NSW, Australia) with an M-Tube (Miltenyi Biotech). For cell lines, the cells were released from the tissue culture vessel with trypsin to isolate a cell pellet. RNA was isolated with Isolate II RNA Mini Kit (Bioline) with on-column DNAse digestion in accordance with the manufacturer’s instructions. Bioanalyzer RNA Nano chip (Agilent, Forrest Hill, Victoria, Australia) was used to assess RNA quality. All samples used for RNA-seq had a RIN (RNA integrity number) of 7 or higher.

### rRNA depletion

5 μg total RNA was depleted of ribosomal and transfer RNA using RiboZero Magnetic Gold Kit (Human/Mouse/Rat)(Illumina, Scoresby, VIC, Australia). Depleted RNA was purified with the Isolate II RNA micro kit (Bioline) and assessed for quality using the Bioanalyzer (Agilent). Depleted RNA was used in library preparation using the NEBNext Ultra RNA Library Prep Kit for Illumina (New England Biolabs, Ipswich, MA #E7560) in accordance with the manufacturer’s instructions. NEBNext Indexed Primers for Illumina (New England Biolabs) were used to barcode samples. Ampure XP beads (Beckman Coulter, Mount Waverley, VIC, Australia) were used for size selection and all purification steps in accordance with the manufacturer’s instructions. 15 cycles of PCR were used for library amplification. Libraries were assessed for quality using a Bioanalyzer High Sensitivity DNA ChIP (Agilent).

### PCR analysis

Genomic DNA was subjected to PCR for a positive control region, S100A8 (S100A8 F: *5*′-GGG TCC CTC GGC ACT TCA-*3*′ and S100A8 *R: 5*′-AAA TCC TGG GGA ATT GGC-*3*′) to ensure that DNA was of sufficient quality for PCR analysis. PCR based detection of HPV DNA was performed using degenerate primers MY09/MY11 and GP5 + /6 + in a nested PCR reaction (MYO9: *5*′-GCM CAG GGW CAT AAY AAT GG-*3,* ′ MY11: *5*′*-*CGT CCM ARR GGA WAC TGA TC-*3*′, GP5+: *5*′*-*TTT GTT ACT GTG GTA GAT ACT AC-*3*′, GP6+: *5*′-GAA AAA TAA ACT GTA AAT CAT ATT C-*3*′). S100A8 and MY09/MY11 PCR was performed in a 50 uL reaction mix using 100 ng of genomic DNA as template, 1x ThermoPol reaction buffer (New England Biolabs), 200 μM dNTP (dATP, dCTP, dGTP, dTTP) (Bioline), 0.2 μM of each oligonucleotide primer and 1.25 U Taq DNA polymerase (New England Biolabs). PCR cycling conditions for S100A8 and MY09/MY11 PCR were 95 °C for 30 seconds, followed by 30 (S100A8) to 40 (MY09/MY11) cycles of 95 °C for 30 seconds, 52 °C for 30 seconds, 68 °C for 30 seconds for 30 cycles and a final extension of 68 °C for 5 minutes. Ten percent of the MY09/MY11 reaction volume was used as a template for the GP5 + /6 + nested PCR. GP5 + /6 + PCR was performed in a 50 mL reaction volume containing 1x PCR reaction buffer I, 0.2 mM dNTP, 2.5 mM magnesium chloride, 0.2 μM of each oligonucleotide primers and 1.25 U AmpliTaq Gold DNA Polymerase. GP5 + /6 + cycling conditions were as follows: 94 °C for 10 minutes, followed by 45 cycles of 94 °C 1 minute, 40 °C 2 minutes, 72 °C 1 minute with a final extension step of 72 °C for 7 minutes.

For HPV testing, each PCR was repeated 3 times and a sample which showed positivity in 1 or more repeats of the GP5 + /6 + PCR was deemed positive.

For S100A8 and MY09/MY11 primer sets Taq Polymerase with Thermopol Buffer (New England Biolabs) was used. After PCR, 10% of the PCR reaction was elotrophoresed on a 2% agarose gel stained with ethidium bromide (Sigma Aldrich) and visualized under UV transillumination.

### Sequencing

Samples were sequenced on the HiSeq 2500 (Illumina) in rapid mode using 2×100bp paired end chemistry.

### Data analysis

The human genome build hg19 genome was downloaded from UCSC. CASAVA (Illumina) was used to demultiplex sequencing reads. The analyses were performed on a high performance computing cluster using PBS Pro 12.01 running on Red Hat Enterprize Linux 6. Viral genomes were downloaded from NCBI on the 1^st^ of April 2015 using search terms Viruses[PORG] and scrdb_refseq[PROP] and combined into a multifasta file. RINS^13^, Readscan^15^ and VirusFinder 2.0^14^ were accessed as per their publications.

Readscan was run using default parameters. RINS was run using default parameters. Bowtie and blast indexes were built using standard parameters. Software versions used were: Bowtie (version 0.12.6)^35^, Trinity (version 050811)^36^, blat (v34)^37^ and NCBI Blast Suite (version 2.2.27)^38^.

VirusFinder2 was run using default parameters. Bowtie2 and blast indexes were built using standard parameters. Software versions used were: NCBI Blast Suite (version 2.2.27)^38^, Bowtie 2 (Version 2.2.5)^35^, BWA (Version 0.6.1)^39^, Trinity (version r2012-06-08)^36^, SVDetect (version 0.8)^40^, Blat (v34)^37^.

For TCGA and TNBC data, bam files were obtained from respective data providers. To minimize data storage and analysis time, sam2fastq (Picard) was used to extract unaligned reads from bam files which were converted to fastq prior to analysis with virus finding software. This approach was first validated using RNA-seq data from HeLa files (SRR702400). For TCGA and TNBC samples, Enterophage PhiX DNA, which is used as a sequencing control on Illumina Hiseq platform, was detected by RINS.

### Reference sequencing samples

RNA Seq datasets were accessed through The Cancer Genome Atlas (TCGA), NCBI Short Read Archive and from^23^. Accession and identification numbers for publicly available datasets are provided in Supplementary Table 1.

### Statistical analysis

Statistical analysis of clinicopathological features of the fresh frozen breast cancer were examined by Chi-squared testing performed in GraphPad Prism (GraphPad Software, Treestar).

## Additional Information

**How to cite this article**: Gannon, O. M. *et al.* No association between HPV positive breast cancer and expression of human papilloma viral transcripts. *Sci. Rep.* **5**, 18081; doi: 10.1038/srep18081 (2015).

## Supplementary Material

Supplementary Table 1

## Figures and Tables

**Table 1:** Frequency of HPV DNA in tissue samples by clinical and histological features.

	All samples N (%)	HPV-positive N (%)	HPV-negative N (%)	*P*-value
Number of samples	90 (100)	14 (16)	76 (84)	
*Age (years)*				
<50	35	7 (50)	27 (36)	0.34
>50	53	7 (50)	47 (61)	
Unknown	2	0 (0)	2 (3)	
*Diagnosis*				
Infiltrating ductal carcinoma	58	9 (64)	49 (64)	0.72
Infiltrating lobular carcinoma	6	1 (7)	5 (7)	
DCIS	3	0 (0)	3 (4)	
Mixed	11	3 (22)	8 (11)	
Benign	10	1 (7)	9 (12)	
Unknown	2	0 (0)	2 (2)	
*Grade*				
1	7	1 (7)	6 (8)	0.84
2	33	5 (36)	28 (37)	
3	35	7 (50)	28 (37)	
DCIS	3	0 (0)	3 (4)	
Unknown	2	0 (0)	2 (3)	
Benign	10	1 (7)	9 (12)	
*Nodes*				
Negative	48	9 (64)	39 (51)	0.53
Positive	30	4 (53)	26 (34)	
Unknown	2	0 (0)	2 (3)	
Benign	10	1 (7)	9 (12)	
*Estrogen Receptor*				
Negative	14	3 (21)	11 (14)	0.62
Positive	64	10 (72)	53 (70)	
Unknown	2	0 (0)	3 (4)	
Benign	10	1(7)	9 (12)	
*Progesterone Receptor*				
Negative	16	3 (21)	13 (17)	0.80
Positive	62	10 (72)	52 (68)	
Unknown	2	0 (0)	2 (3)	
Benign	10	1 (7)	9 (12)	
*HER2-neu*				
Negative	63	11 (79)	52 (68)	0.85
Positive	13	2 (14)	11 (14)	
Unknown	4	0 (0)	4 (5)	
Benign	10	1 (7)	9 (12)	
*Triple negative status*				
Triple Negative	6	1 (7)	5 (7)	1
Non-triple negative	70	12 (86)	58 (76)	
Unknown	4	0 (0)	4 (5)	
Benign	10	1 (7)	9 (12)	

HPV is Human papilloma virus, a positive sample is positive in 1 or greater of 3 MY09/MY11 nested PCR technical repeats. *P*-Values were obtained by Chi Squared test and a P value of less than 0.05 is statistically significant. Unknown and benign samples were not included in Chi-squared testing for association of HPV DNA with clinic-pathological characteristics.

**Table 2:** Clinicopathological Features of tissue samples that were HPV positive.

ID	RINS	Readscan	VF	Pathology	Age	Grade	Nodes	Size (mm)	ER	PR	Her2
HeLa	HPV18	HPV18	HPV18								
43	**n.d**	**n.d**	**n.d**	IDC	40	3	POS	22	POS	POS	NEG
PS	**n.d**	**n.d**	**n.d**	IDC	51	2	NEG	25	POS	POS	NEG
SM	**n.d**	**n.d**	**n.d**	IDC + DCIS	30	2	POS	18	POS	POS	NEG
PH	**n.d**	**n.d**	**n.d**	IDC	44	3	POS	15	NEG	NEG	NEG
MG	**n.d**	**n.d**	**n.d**	IDC	53	3	NEG	15	NEG	NEG	POS
WB	n.a	n.a	n.a	IDC	48	2	NEG	5	POS	POS	NEG
2	n.a	n.a	n.a	IDC	68	3	POS	30	NEG	NEG	POS
55	n.a	n.a	n.a	IDC	69	3	NEG	14	POS	POS	NEG
51	n.a	n.a	n.a	IDC	62	3	NEG	10	POS	POS	NEG
KM	n.a	n.a	n.a	IDC + DCIS	35	3	NEG	20 + 20 DCIS	POS	POS	NEG
54	n.a	n.a	n.a	IDC	60	1	NEG	12	POS	POS	NEG
TP	n.a	n.a	n.a	IDC + DCIS	31	2	NEG	18 + 20 DCIS	POS	POS	NEG
53	n.a	n.a	n.a	ILC	60	2	NEG	25	POS	POS	NEG
42	n.a	n.a	n.a	FA	40						

14 tissue samples were positive for HPV DNA by nested PCR. Samples in bold were analyzed by RNA seq, samples below the dotted line were not analyzed by RNA seq. RINS is Rapid Identification of non human sequences bioinformatic pipeline, READSCAN is readscan bioinformatic pipeline, VF is Virusfinder 2.0 bioinformatic pipeline, ER is estrogen receptor, PR is progesterone receptor, HER2 is human epidermal growth factor receptor 2, HPV18 is human papilloma virus 18, n.d. is no virus detected in bioinformatic analysis, POS is positive, NEG is negative, IDC is infiltrating ductal carcinoma, DCIS is ductal carcinoma *in situ*, ILC is infiltrating lobular carcinoma, FA is fibroadenoma, n.a. is not available.

**Table 3:** Positive control RNA seq data sets were analysed by RINS for presence of viral transcripts.

Reference	Sample	Library	Platform	Read	Virus	Viral reads	Total reads	ppm
SRR540252	HeLa	Poly A	N.A	Paired	HPV18	21407	8000000	2675
SRR702400	HeLa	rRNA	GAII	Single	HPV18	26378	21000000	1256
SRR629571	HeLa	N.A	HiSeq	Paired	HPV18	44405	15000000	2960
SRR073726	Prostate	Poly A	N.A	Single	HPV18	25755	13000000	1981
SRR069060	Akata	N.A	GAII	Single	HHV4	34696	26000000	949
SRR497704	CD4 T-cells	N.A	GAII	Single	HIV	64478	23000000	2803
UNCID-1487840	HNSCC	Poly A	HiSeq	Paired	HPV16	3470	182000000	19
UNCID-1488824	HNSCC – normal adjacent	Poly A	Hiseq	Paired	n.d	0	169000000	0
UNCID-1494200	HNSCC	Poly A	HiSeq	Paired	HPV33	29131	112000000	260
UNCID-1489199	HNSCC – normal adjacent	Poly A	HiSeq	Paired	n.d	0	157000000	0

Reference describes the accession identification used for data download from NCBI (SRR) or TCGA (UNCID). Poly A is a library prepared from poly A isolated mRNA, rRNA is a library prepared from rRNA depleted library. Sample describes the cell line or sample type used in the analysis, where HNSCC is head and neck squamous cell carcinoma. GAII is Illumina Genome Analyzer II, HiSeq refers to HiSeq 1000, 2000 or 2500 platforms. HPV is human papilloma virus, HHV is human herpes virus 4, HIV is human immunodeficiency virus. Total reads are total number of next generation sequencing reads, Viral reads are number of viral reads aligning to viral sequence. N.A is not available, n.d. is not detected, ppm is read proportion per million.
