# Supplementary material for: No association between HPV positive breast cancer and expression of human papilloma viral transcripts
Source: Sci Rep. 2015 Dec 14;5:18081. doi: 10.1038/srep18081 (PMC4677295; doi:10.1038/srep18081)
Supplement: Supplementary Table 1 [file srep18081-s1.doc]

No association between HPV positive breast cancer and expression of human papilloma viral transcripts

Orla Margaret Gannon ^1^, Annika Antonsson ^2^, Michael Milevskiy ^3^, Melissa A Brown ^3,4^, Nicholas Andrew Saunders ^+~^ ^1^**,** Ian C Bennett ^+^ * ^5,6^

**Affiliations:**

1. The University of Queensland Diamantina Institute, The University of Queensland, Translational Research Institute, Brisbane, QLD

2. Department of Population Health, QIMR Berghofer Medical Research Institute, Herston, QLD

3. School of Chemistry and Molecular Biosciences, The University of Queensland, Brisbane, QLD

4. Faculty of Medicine and Biomedical Sciences, The University of Queensland, Brisbane, QLD

5. Princess Alexandra Hospital, University of Queensland Department of Surgery, Brisbane, QLD

6. Wesley Private Hospital, Auchenflower, Brisbane, QLD

+ Co-corresponding author

~ Correspondence to: A/Prof Nicholas Saunders, Epithelial Pathobiology Group, The Unversity of Queensland Diamantina Institute, The University of Queensland, Translational Research Institute, 37 Kent Street, Woolloongabba, QLD 4102. Phone: +61 (0)7 3443 7091, E-mail: [nsaunders@uq.edu.au](mailto:nsaunders@uq.edu.au)

* Correspondence to: A/Prof Ian Bennett, Breast and Endocrine Surgeon, 101 Wickham Terrace, Spring Hill, QLD 4000. Phone: +61 (0)7 38390006; E-mail: [bensurg@bigpond.net.au](mailto:bensurg@bigpond.net.au)

**Short title:** HPV transcripts are not found in human breast cancer

Supplementary Data

| **Identifier** | **Specimen** | **Virus** | **Hormone** | **Histology** | **Age** | **Stage** | **Nodes** |
| --- | --- | --- | --- | --- | --- | --- | --- |
| SA018 | TNBC | n.d | TNBC | IDC | 49 | n/a | Positive |
| SA051 | TNBC | n.d | TNBC | IDC | 51 | n/a | Positive |
| SA052 | TNBC | n.d | TNBC | other | 86 | n/a | Positive |
| SA053 | TNBC | n.d | TNBC | IDC | 53 | n/a | Negative |
| SA055 | TNBC | n.d | TNBC | IDC | 71 | n/a | Positive |
| SA102 | TNBC | n.d | TNBC | n/a | n/a | n/a | n/a |
| SA219 | TNBC | n.d | TNBC | IDC | 58 | n/a | Positive |
| SA233 | TNBC | n.d | TNBC | IDC | 58 | n/a | Negative |
| SA092 | TNBC | n.d | TNBC | IDC | n/a | n/a | n/a |
| SA217 | TNBC | n.d | TNBC | IDC | 51 | n/a | Negative |
| SA228 | TNBC | n.d | TNBC | IDC | 58 | n/a | Positive |
| SA229 | TNBC | n.d | TNBC | mixed | 43 | n/a | Negative |
| SA231 | TNBC | n.d | TNBC | IDC | 25 | n/a | Positive |
| SA235 | TNBC | n.d | TNBC | IDC | 39 | n/a | Positive |
| SA029 | TNBC | n.d | TNBC | IDC | 60 | n/a | Positive |
| SA054 | TNBC | n.d | TNBC | IDC | 63 | n/a | Negative |
| SA068 | TNBC | n.d | TNBC | IDC | 69 | n/a | Positive |
| SA073 | TNBC | n.d | TNBC | IDC | 55 | n/a | Negative |
| SA074 | TNBC | n.d | TNBC | IDC | 66 | n/a | Positive |
| SA075 | TNBC | n.d | TNBC | IDC | 49 | n/a | Positive |
| SA083 | TNBC | n.d | TNBC | IDC | 37 | n/a | Positive |
| SA097 | TNBC | n.d | TNBC | IDC | 40 | n/a | n/a |
| SA103 | TNBC | n.d | TNBC | IDC | 61 | n/a | n/a |
| SA105 | TNBC | n.d | TNBC | IDC | 37 | n/a | n/a |
| SA106 | TNBC | n.d | TNBC | IDC | 78 | n/a | n/a |
| SA208 | TNBC | n.d | TNBC | IDC | 37 | n/a | Negative |
| SA210 | TNBC | n.d | TNBC | IDC | 55 | n/a | Positive |
| SA211 | TNBC | n.d | TNBC | IDC | 34 | n/a | Positive |
| SA213 | TNBC | n.d | TNBC | IDC | 49 | n/a | Negative |
| SA220 | TNBC | n.d | TNBC | IDC | 37 | n/a | Negative |
| SA223 | TNBC | n.d | TNBC | IDC | 48 | n/a | Positive |
| SA230 | TNBC | n.d | TNBC | IDC | 66 | n/a | Negative |
| SA232 | TNBC | n.d | TNBC | IDC | 59 | n/a | Negative |
| SA227 | TNBC | n.d | TNBC | IDC | 61 | n/a | Negative |
| ERR030883 | Normal | n.d | n/a | n/a | n/a | n/a | n/a |
| TCGA-A7-A13E-01A-11R-A12P-07 | Normal | n.d | n/a | n/a | 62 | n/a | n/a |
| TCGA-A7-A13E-11A-61R-A12P-07 | BRCA | n.d | ER+ HER2+ PR - | IDC | 62 | IIB | Positive |
| TCGA-A7-A13G-01A-11R-A13Q-07 | BRCA | n.d | ER+ PR+ HER2 - | IDC | 79 | IIA | Negative |
| TCGA-BH-A0AZ-11A-22R-A12P-07 | Normal | n.d | n/a | n/a | 47 | n/a | n/a |
| TCGA-AC-A23H-01A-11R-A157-07 | BRCA | n.d | ER+ PR- HER2+ | IDC | 90 | IIA | n/a |
| TCGA-A7-A0DB-11A-33R-A089-07 | Normal | n.d | n/a | n/a | 56 | n/a | n/a |
| TCGA-AC-A2FF-01A-11R-A17B-07 | BRCA | n.d | ER+ PR+ | ILC | 40 | IIB | Positive |
| TCGA-A8-A09M-01A-11R-A00Z-07 | BRCA | n.d | ER+ PR+ HER2- | IDC | 75 | IIIC | n/a |
| TCGA-A7-A0DB-01A-11R-A00Z-07 | BRCA | n.d | ER+ PR+ HER2- | IDC | 56 | IIA | Negative |
| TCGA-AC-A2FM-11B-32R-A19W-07 | Normal | n.d | n/a | n/a | 87 | n/a | n/a |
| TCGA-A7-A13F-01A-11R-A12P-07 | BRCA | n.d | ER+ PR+ HER2+ | IDC | 44 | IIIA | Positive |
| TCGA-AC-A2FF-11A-13R-A17B-07 | Normal | n.d | n/a | n/a | 40 | n/a | n/a |
| TCGA-BH-A0AZ-01A-21R-A12P-07 | BRCA | n.d | ER+ PR+ HER2- | IDC | 47 | IIIA | Positive |
| TCGA-AC-A23H-11A-12R-A157-07 | Normal | n.d | n/a | n/a | 90 | n/a | n/a |
| TCGA-A7-A425-01A-11R-A24H-07 | BRCA | n.d | ER+ PR+ HER2+ | ILC | 70 | IIIC | Positive |
| TCGA-A7-A26J-01A-11R-A169-07 | BRCA | n.d | ER+ PR+ HER2 - | IDC | 49 | IIA | Negative |
| TCGA-AC-A2FB-01A-11R-A17B-07 | BRCA | n.d | ER+ PR+ HER2+ | ILC | 65 | IIA | Negative |
| TCGA-AC-A2FM-01A-11R-A19W-07 | BRCA | n.d | n/d | ILC | 87 | IIB | Negative |
| TCGA-A8-A06Q-01A-11R-A034-07 | BRCA | n.d | ER+ PR+ HER2- | IDC | 63 | IIIA | n/a |
| TCGA-A7-A13F-11A-42R-A12P-07 | Normal | n.d | n/a | n/a | 44 | n/a | n/a |
| TCGA-AC-A2FB-11A-13R-A17B-07 | Normal | n.d | n/a | n/a | 65 | n/a | n/a |
| TCGA-A2-A0SW-01A-11R-A084-07 | BRCA | n.d | ER+ PR- | IDC | 82 | IV | Positive |
| TCGA-A8-A06T-01A-11R-A00Z-07 | BRCA | n.d | ER+ PR+ HER2 + | IDC | 75 | IIIA | n/a |
| TCGA-A2-A0YI-01A-31R-A10J-07 | BRCA | n.d | ER+ PR+ HER2 - | IDC | 62 | IA | Negative |
| TCGA-A7-A13G-11A-51R-A13Q-07 | Normal | n.d | n/a | n/a | 79 | n/a | n/a |
| TCGA-A2-A0ET-01A-31R-A034-07 | BRCA | n.d | ER+ PR+ HER2- | IDC | 58 | IIIA | Positive |
| TCGA-A8-A09R-01A-11R-A00Z-07 | BRCA | n.d | ER+ PR+ HER2- | IDC | 82 | IIB | n/a |
| TCGA-A2-A25B-01A-11R-A169-07 | BRCA | n.d | ER+ PR+ HER2- | IDC | 39 | IIB | Positive |

**Supplementary Table 1**. **Summary of TCGA and TNBC data sets analysed by RINS for presence of viral transcripts**. Identifier is unique identifier/barcode which can be used to identify the sample studied, TNBC is triple negative breast cancer (from Shah *et al.*), BRCA is breast cancer from The Cancer Genome Atlas, n.d. is virus not detected. n/a is not available, ER is estrogen receptor, PR is progesterone receptor, HER2 is HER2neu, + is positive, - is negative, IDC is infiltrating ductal carcinoma, ILC is infiltrating lobular carcinoma, mixed is IDC and ILC, other is pathology not defined. If hormone status is not listed it was not described in TCGA datset. Positive indicates lymph nodes with tumour detectable on histology. 10 normal breast tissues and 53 breast cancers were analysed
